# Variability of non-Gaussian diffusion MRI and intravoxel incoherent motion (IVIM) measurements in the breast

**DOI:** 10.1371/journal.pone.0193444

**Published:** 2018-03-01

**Authors:** Mami Iima, Masako Kataoka, Shotaro Kanao, Makiko Kawai, Natsuko Onishi, Sho Koyasu, Katsutoshi Murata, Akane Ohashi, Rena Sakaguchi, Kaori Togashi

**Affiliations:** 1 Department of Diagnostic Imaging and Nuclear Medicine, Kyoto University Graduate School of Medicine, Kyoto, Japan; 2 The Hakubi Center for Advanced Research, Kyoto University, Kyoto, Japan; 3 Siemens Healthcare K.K., Shinagawa, Tokyo, Japan; Mathematical Institute, HUNGARY

## Abstract

We prospectively examined the variability of non-Gaussian diffusion magnetic resonance imaging (MRI) and intravoxel incoherent motion (IVIM) measurements with different numbers of b-values and excitations in normal breast tissue and breast lesions. Thirteen volunteers and fourteen patients with breast lesions (seven malignant, eight benign; one patient had bilateral lesions) were recruited in this prospective study (approved by the Internal Review Board). Diffusion-weighted MRI was performed with 16 b-values (0–2500 s/mm^2^ with one number of excitations [NEX]) and five b-values (0–2500 s/mm^2^, 3 NEX), using a 3T breast MRI. Intravoxel incoherent motion (flowing blood volume fraction [fIVIM] and pseudodiffusion coefficient [D*]) and non-Gaussian diffusion (theoretical apparent diffusion coefficient [ADC] at *b* value of 0 sec/mm^2^ [ADC_0_] and kurtosis [K]) parameters were estimated from IVIM and Kurtosis models using 16 b-values, and synthetic apparent diffusion coefficient (sADC) values were obtained from two key b-values. The variabilities between and within subjects and between different diffusion acquisition methods were estimated. There were no statistical differences in ADC_0_, K, or sADC values between the different b-values or NEX. A good agreement of diffusion parameters was observed between 16 b-values (one NEX), five b-values (one NEX), and five b-values (three NEX) in normal breast tissue or breast lesions. Insufficient agreement was observed for IVIM parameters. There were no statistical differences in the non-Gaussian diffusion MRI estimated values obtained from a different number of b-values or excitations in normal breast tissue or breast lesions. These data suggest that a limited MRI protocol using a few b-values might be relevant in a clinical setting for the estimation of non-Gaussian diffusion MRI parameters in normal breast tissue and breast lesions.

## Introduction

Diffusion magnetic resonance imaging (MRI) has been shown to be an important diagnostic biomarker for tumor characterization [1], including assessment of breast tumors [2] or treatment responses [3], without the requirement for contrast agents. Although the majority of studies have estimated apparent diffusion coefficient (ADC) values from diffusion-weighted imaging (DWI) data, several other diffusion analysis methods have been proposed using multiple b-values in the breast [4] and other organs [5, 6].

Intravoxel incoherent motion (IVIM), which can estimate perfusion at the microcapillary level [7], combined with non-Gaussian diffusion MRI models such as Kurtosis, may provide additional information on water diffusion in breast tissue compared with ADC. Indeed, several studies have examined the clinical utility of these techniques for differentiation of malignant and benign breast tumors [4, 8] and correlations with prognostic factors [8]. Further, synthetic ADC (sADC), which is obtained only from two key b-values [9], can encompass both non-Gaussian and Gaussian diffusion effects compared with the standard ADC diffusion model, without additional scanning time.

One of the challenging features for diffusion MRI is the low signal-to-noise ratio (SNR) observed at high b-values and the recommended increase in the number of excitations (NEX) to generate valid diffusion MR data [10]. Optimization of diffusion anisotropy measurements protocols was reported using a phantom and brain tissue [11]. However, the optimal diffusion MRI acquisition parameters (i.e., b-values and NEX) in the breast have not been standardized. As such, a range of combinations of high b-values (600–3000 s/mm^2^) have been reported, and together with the non-Gaussian diffusion effect in tissues, this results in variability in calculated ADC values [10]. Thus, it is difficult to compare findings between various reports and multicenter studies, especially for estimation of non-Gaussian diffusion parameters [4, 12, 13].

The associations of ADC values with hormonal status in normal breast parenchyma [14–16] and anisotropy values in volunteers [17] were previously reported. However, the behavior of non-Gaussian DWI and IVIM parameters with different MRI protocols or in lesions remains unknown. This is particularly important, as changes in these parameters with different protocols or tissues may change the non-Gaussian DWI and IVIM measurements. Thus, the aim of the present study was to prospectively examine the optimal NEX and number of b-values of DWI in normal breast tissue and breast lesions.

## Materials and methods

### Participant population

This study was approved by the institutional review board of Kyoto University, and was conducted in accordance with the ethical standards of the World Medical Association (Declaration of Helsinki), with written informed consent from all participating subjects. Thirteen women (32.2 ± 6.8 years, range: 19–43 years) were consequently recruited to the study between July 2014 and March 2015. No subjects were on an extended-cycle contraception regimen. Volunteers were scanned between the 5^th^ and 15^th^ day of their menstrual cycles. Fourteen patients with breast lesions (seven malignant, eight benign; one patient had bilateral lesions; 52.4 ± 12.8 years of age, range: 23–75 years) were also included in the study in December 2017. Malignant lesions included six invasive ductal carcinomas and one mucinous carcinoma. Benign lesions included eight fibroadenomas. One patient had bilateral breast tumors, with invasive ductal carcinoma in the left breast and fibroadenoma in the right breast. The mean tumor diameter was 38.7 mm (range: 16–120 mm) in malignant lesions, and 11.5 mm (8–22 mm) in benign lesions. All lesions were histopathologically or clinicoradiologically confirmed.

### Phantom preparation

A cylindrical alkane (nonane) phantom (9 cm diameter) was used to estimate the behaviors of the signals and diffusion parameters. This phantom was developed for calculation of the noise correction factor [4]. The region-of-interest (ROI) was located at the center of the nonane phantom, and the diffusion parameters were obtained. ADC_0_ (theoretical apparent diffusion coefficient at *b* value of 0 sec/mm^2^) values were used to examine the effect of averaging on the diffusion parameters. The room temperature was set at 24°C.

### MRI acquisitions

Breast MRI was performed using a 3T system (MAGNETOM Trio, A Tim System; Siemens Healthcare, Erlangen, Germany) equipped with a dedicated 16-channel breast array coil for volunteers. Patients and a phantom were scanned with 3T system (MAGNETOM Prisma; Siemens Healthcare) equipped with a dedicated 18-channel breast array coil. The following images were obtained after localizers were acquired:

1. Bilateral fat-suppressed T2-weighted images (repetition time/echo time 5500/77 [Trio] and 70 [Prisma] ms, flip angle 140°, turbo factor 20, field of view 330 × 330 mm^2^, matrix 448 × 448, slice thickness 3 mm, acquisition time 1 min 30 s).
2. Trace-weighted diffusion images (single shot EPI [echo-planar imaging]; WIP [work in progress] sequence) with spectral attenuated inversion recovery (SPAIR) for fat suppression with the following parameters: volunteers had 16 b-value (A) and five b-value (B) datasets; patients had five b-value (B) datasets.
  (A) Sixteen b-values (3, 5, 10, 20, 30, 50, 70, 100, 200, 400, 600, 800, 1000, 1500, 2000, and 2500 s/mm^2^; the minimum b-value was 3 s/mm^2^ because of the presence of crusher pulses on both sides of the refocus pulse), one NEX, and a scan time of 3 min 55 s.
  (B) Five b-values (3, 100, 200, 1500, and 2500 s/mm^2^), three NEXs, and a total scan time of 3 min 30 s. These parameters were determined to balance the acquisition time of the 16 b-value and five b-value DWI datasets.
  Five b-values were selected to include key b-values (200 and 1500 s/mm^2^) in the breast, the minimum and maximum value of the 16 b-values (3 and 2500 s/mm^2^), and the intermediate values between 0 and 200 s/mm^2^ (100) for the estimation of IVIM parameters. The images with one NEX were extracted from the first of three NEX datasets for the analysis of the five b-value DWI dataset.
3. For both protocols, the common acquisition parameters were as follows: repetition time/echo time 4600/86 ms, field of view 160 × 300 mm^2^, matrix 80 × 166, slice thickness 3.0 mm, 25 slices without a gap, bandwidth 1585 Hz, and generalized autocalibrating partial parallel acquisition (GRAPPA) with an acceleration factor of 2.

### Data processing

The signals were processed in two steps: (1) estimating the diffusion component, *Fdiff*, using the kurtosis diffusion model corrected for noise bias [4]; and (2) estimating the perfusion component, *Fperf*, against the residual signal, after the diffusion component has been removed, as follows:

$$M(b)={\left[S{\left(b\right)}^{2}+NCF\right]}^{1/2}$$ (1)

S(b)=fIVIMFperf+(1−fIVIM)Fdiff (2)

where M(b) is the overall measured signal, f_IVIM_ is the volume fraction of incoherently flowing blood in the tissue, and NCF is the noise correction factor, which characterizes the ‘intrinsic’ noise contribution from the data points.

The signal attenuation curve, M(b), against the b-values between 200 and 2500 s/mm^2^ was fitted using the kurtosis diffusion model to estimate ADC_0_ and K (kurtosis):

$$Fdiff=exp[-b{ADC}_{0}+{\left({bADC}_{0}\right)}^{2}K/6]$$ (3)

The diffusion component was then subtracted from the signal, and the remaining signal for b-values <200 s/mm^2^ was fitted using the IVIM model to obtain the estimates of the flowing blood fraction, f_IVIM_, and pseudo-diffusion coefficient, D*, as follows:

fIVIMFperf=S(b)−(1−fIVIM)Fdiff (4)

$$Fperf=exp(-bD^{*})$$ (5)

sADC, encompassing both non-Gaussian and Gaussian effects [9], was calculated as:

$$sADC=ln[{{\left(Sn(Lb\right)}^{2}-NCF)}^{1/2}/{{\left(Sn(Hb\right)}^{2}-NCF)}^{1/2}]/(Hb-Lb)$$ (6)

where Lb is the low key b-value, and Hb is the high key b-value ([Lb, Hb] = [200, 1500]).

This sADC, which was introduced to handle non-Gaussian and Gaussian diffusion effects, requires only two key b-values for estimation, and can be obtained using a similar scanning time as for standard ADC with two b values (e.g., b = 0 and 1000 s/mm^2^).

This process was performed using the nonlinear subspace trust region fitting algorithm built into Matlab (Mathworks, Natick, MA, USA) at both the ROI level and the pixel level to generate the parametric maps of diffusion parameters. sADC values were also generated at the pixel level from the nonane phantom to examine the effects of NEX on diffusion signals and the derived parameters. Nonane is known to have diffusion coefficients similar to ADC in the breast [18], and we previously reported the detailed setup for scanning a nonane phantom [4].

### Regions of interest

The DWI image slice containing the largest amount of fibroglandular tissue for volunteers and lesion for patients was selected, and ROI was manually drawn for each case by a radiologist A (M.I.) with 9 year experience in breast MRI. (Strong intraclass correlation coefficients [ICC] in ADC_0_, K, and sADC or moredate ICC in f_IVIM_ values between radiologist A and radiologist B [M.K.] are previously reported on DWI of breast lesions [19]).

### Statistical analysis

Coefficients of variation were calculated to evaluate the variability of non-Gaussian diffusion and IVIM parameters. The between-subject coefficient of variation (bCV) for each parameter was estimated as the ratio of the standard deviation to the mean for a single variable. The within-subject CV (wCV), expressed as a percentage, was calculated as the ratio of the within-subject standard deviation to the overall mean of the estimates from two of the three different DWI acquisition methods [20].

Firstly, significant differences among diffusion parameters obtained with the different diffusion acquisition methods were compared using Wilcoxon tests (paired samples). Then, ICCs with average measures were calculated to estimate the agreement of these diffusion parameters between the different diffusion acquisition methods. The agreement was defined as almost perfect (0.8–1.0), substantial (0.6–0.8), moderate (0.4–0.6), and poor (<0.4). Bonferroni correction was performed for multiple comparison. *P* value less than .017 (calculated with equation .05/3) and less than .01 (calculated with equation .05/5) were used to indicate the significance when testing the three and five diffusion parameters, respectively. bCVs and wCVs were calculated using Excel (Microsoft Excel 2013; Redmond, Washington, U.S.). Wilcoxon tests and ICCs were performed using statistical software (Medcalc version 11.3.2.0; Ostend, Belgium).

## Results

### Phantom

The distributions of the ADC_0_ values in a nonane phantom (to show the effects of averaging) are shown in S1 Fig. The CVs of the ADC_0_ values decreased as the NEX increased (0.470% for one NEX, 0.242% for two NEX, and 0.229% for three NEX.).

### Volunteers and patients

**Fig 1:**
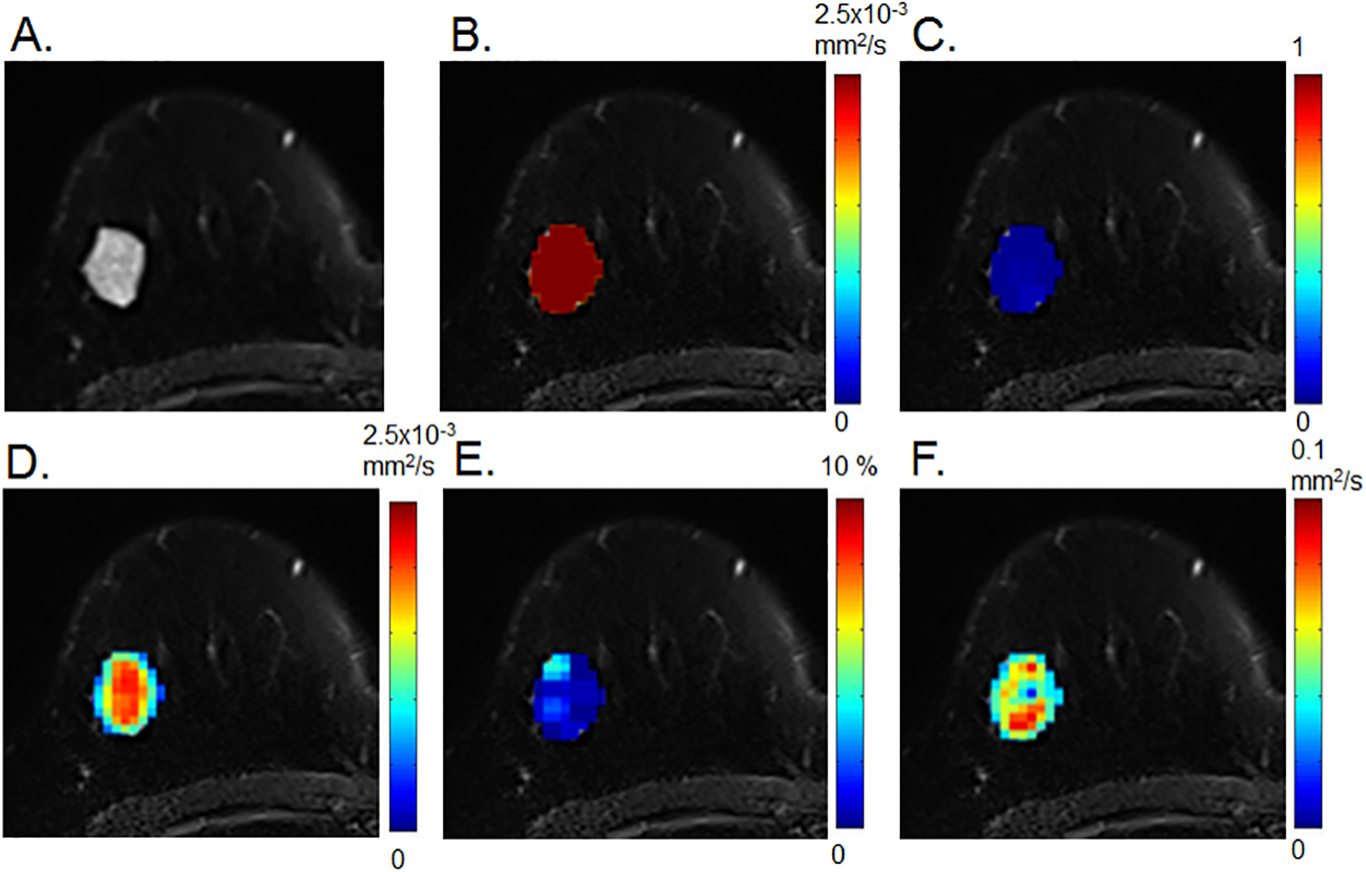
Diffusion weighted imaging (DWI) and intravoxel incoherent motion (IVIM) parametric maps of fibroadenoma in a 63-year-old woman. Axial diffusion and IVIM magnetic resonance imaging (MRI) maps were overlaid on T2-weighted images. (A) T2-weighted image, (B) ADC_0_ map, (C) K map, (D) sADC map, (E) fIVIM map, and (F) D* map.

**Fig 2:**
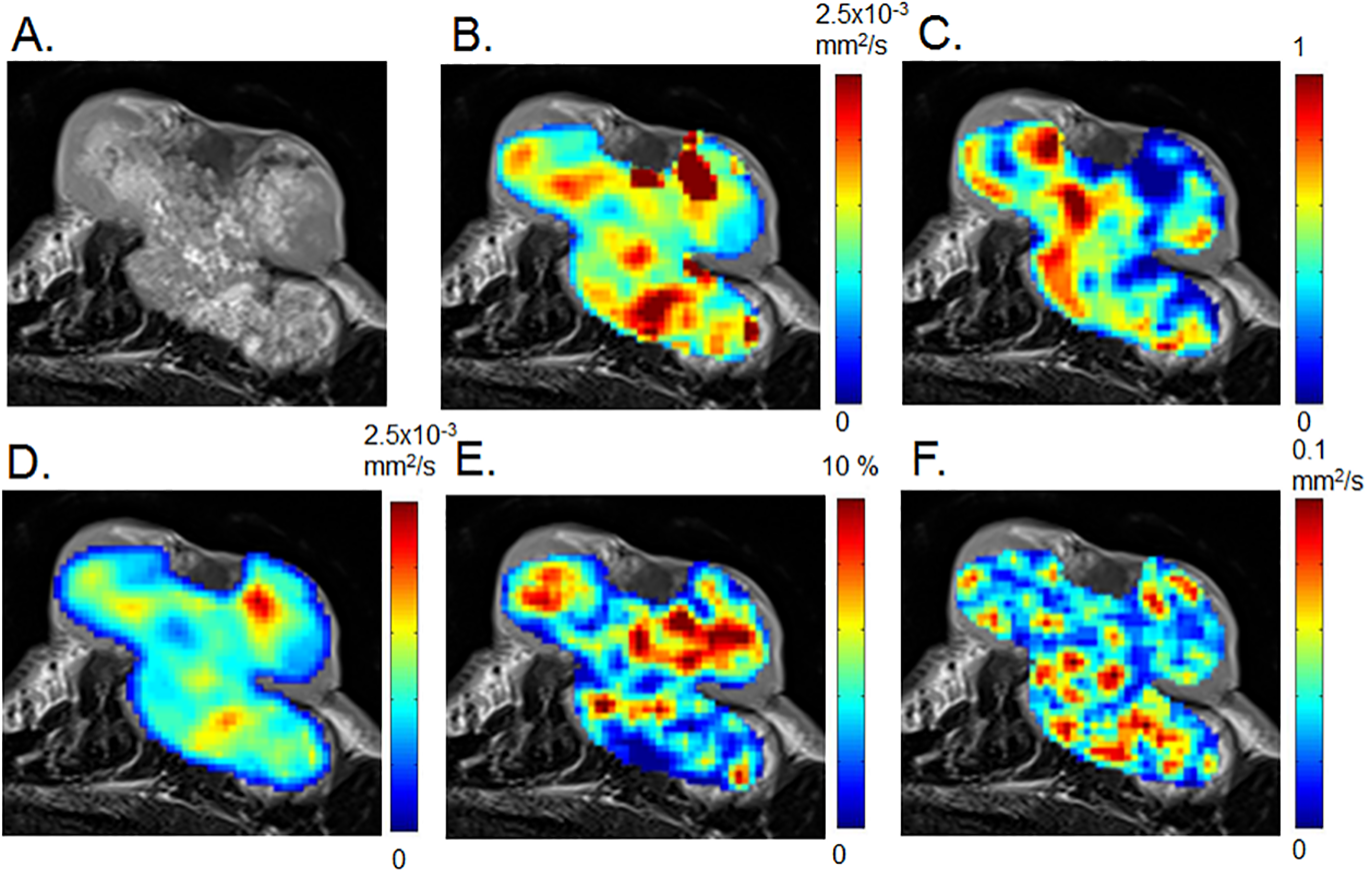
DWI and IVIM parametric maps of invasive ductal carcinoma in a 63-year-old woman. The patient had bilateral lesions involving invasive ductal carcinoma in the left breast and fibroadenoma in the right breast (Fig 1). Axial diffusion and IVIM MRI maps were overlaid on T2-weighted images. (A) T2-weighted image, (B) ADC_0_ map, (C) K map, (D) sADC map (E) fIVIM map, (F) D* map.

An example of non-Gaussian DWI and IVIM parametric maps in malignant and benign breast tumors from five b-value datasets with one NEX is shown in Figs 1 and 2. There were marked differences in the distribution of diffusion and perfusion parameters between malignant and benign lesions. There was a homogenous distribution of high ADC_0_ and sADC, and of low K and fIVIM, in fibroadenoma (Fig 1).

By contrast, there was a more heterogeneous pattern of lower ADC_0_ and sADC values, and of higher fIVIM and K values, in invasive ductal carcinoma (Fig 2).

Diffusion and perfusion parameters as well as bCVs in normal breast tissues are provided in Table 1. Individual diffusion and perfusion parameters in normal breast tissues are shown in S1 Table. The wCVs and *P* values comparing the diffusion and perfusion parameters obtained from different DWI datasets in normal breast tissues are shown in Table 2. There were no statistical differences in the diffusion parameters between the 16 b-values, five b-values with one NEX, and five b-values with three NEX in normal breast tissue. The wCVs in diffusion and perfusion parameters were smaller than the bCVs. ADC_0_ and sADC tended to have smaller bCV and wCV than other parameters.

**Table 1:** Diffusion and perfusion parameters and between-subject coefficients of variations (bCV) in normal breast tissues.

Parameters	16 b values (1 NEX)	5 b values (1 NEX)	5 b values (3 NEX)
**ADC**_**0**_ **(10**^**−3**^ **mm**^**2**^**/s)**	2.32 ± 0.20 (0.085)	2.41 ± 0.37 (0.152)	2.39 ± 0.43 (0.178)
**K**	0.18 ± 0.18 (0.999)	0.13 ± 0.21 (1.591)	0.16 ± 0.26 (1.671)
**fIVIM (%)**	0.26 ± 0.64 (2.428)	0.74 ± 1.01 (1.377)	1.17 ± 2.42 (2.068)
D* (10^**−3**^ **mm**^**2**^**/s)**	70.6 ± 46.0 (0.652)	80.0 ± 38.2 (0.478)	67.6 ± 43.1 (0.637)
**sADC (10**^**−3**^ **mm**^**2**^**/s)**	2.11 ± 0.32 (0.153)	2.13 ± 0.38 (0.178)	2.10 ± 0.43 (0.206)

Mean values and standard deviations are shown (n = 13). Between-subject coefficients of variation (bCV) are shown in parentheses.

**Table 2:** Within-subject coefficient of variation (wCV) and *P* values comparing the normal breast tissue datasets using different combinations of b-values and NEXs.

Parameters	16 b (1 NEX) vs. 5 b (1 NEX)		16 b (1 NEX) vs. 5 b (3 NEX)		5 b (1 NEX) vs. 5 b (3 NEX)	
	wCV	*P* value	wCV	*P* value	wCV	*P* value
**ADC**_**0**_	2.04%	0.17	2.65%	0.34	2.35%	0.74
**K**	13.9%	0.13	17.4%	0.41	18.1%	1.00
**fIVIM**	45.6%	0.38	73.5%	0.41	55.9%	1.00
D*	14.7%	0.65	18.1%	0.68	11.4%	0.23
**sADC**	2.62%	0.31	2.63%	0.74	2.66%	0.68

*P* values for Wilcoxon tests are shown.

Table 3 shows diffusion and perfusion parameters with their bCVs in malignant and benign breast tumors. Their wCVs and *P* values comparing the diffusion and perfusion parameters using different b values or NEXs in malignant and benign breast tumors are provided in Table 4.

**Table 3:** Diffusion and perfusion parameters in the datasets of malignant and benign breast tumors.

Parameters	malignant (5 b values with 1 NEX)	malignant (5 b values with 3 NEX)	benign (5 b values with 1 NEX)	benign (5 b values with 3 NEX)
**ADC**_**0**_ **(10**^**−3**^ **mm**^**2**^**/s)**	1.20 ± 0.43 (0.359)	1.16 ± 0.39 (0.332)	2.33 ± 0.41 (0.174)	2.41 ± 0.36 (0.150)
**K**	0.80 ± 0.18 (0.220)	0.84 ± 0.18 (0.211)	0.20 ± 0.28 (1.402)	0.21 ± 0.29 (1.397)
**fIVIM (%)**	7.48 ± 1.93 (0.258)	8.35 ± 2.30 (0.275)	3.96 ± 3.81 (0.961)	4.38 ± 4.34 (0.992)
D* (10^**−3**^ **mm**^**2**^**/s)**	32.1 ± 31.8 (0.990)	24.3 ± 18.1 (0.746)	35.5 ± 41.3 (1.165)	68.6 ± 43.5 (0.635)
**sADC (10**^**−3**^ **mm**^**2**^**/s)**	0.87 ± 0.25 (0.287)	0.85± 0.25 (0.293)	2.04 ± 0.59 (0.290)	2.08 ± 0.59 (0.283)

Mean values and standard deviations are shown in seven malignant and eight benign lesions. bCVs are shown in parentheses.

**Table 4:** wCV and *P* values comparing the malignant and benign breast tumor datasets using one and three NEXs of five b-values.

Parameters	malignant		benign	
	wCV	*P* value	wCV	*P* value
**ADC**_**0**_	1.82%	0.38	2.15%	0.31
**K**	2.71%	0.58	1.74%	0.31
**fIVIM**	8.38%	0.38	16.8%	0.84
D*	33.4%	1.00	34.0%	0.11
**sADC**	1.40%	0.30	2.54%	0.25

*P* values for Wilcoxon tests are shown.

There were no statistical differences in the diffusion parameters between the five b-values with one NEX and five b-values with three NEX in the malignant or benign breast lesions. Larger bCVs were observed in K and perfusion parameters compared with diffusion coefficient parameters in normal breast tissue and benign lesions. The bCVs in diffusion and perfusion parameters were comparable (except for D*) in malignant lesions.

The decrease in wCVs compared with bCVs was remarkable both in malignant and benign breast lesions. The wCV of fIVIM tended to be smaller in breast lesions compared to normal breast tissue, while D* tended to have larger wCV values in lesions compared to normal breast tissue.

The agreement of diffusion and perfusion parameters are provided in normal breast tissues (Table 5) and lesions (Table 6). Overall, there was almost perfect agreement of ADC_0_, K, and sADC values between the different numbers of b-values (16 b-values or five b-values) or excitations (three or one NEX), except for a substantial agreement of ADC_0_ values between 16 b-values (one NEX) and five b-values (three NEX) in normal breast tissue (Table 5).

**Table 5:** Intraclass correlation coefficient of diffusion parameters comparing the normal breast tissue datasets using different combinations of b-values.

Parameters	16 b (1 NEX) vs. 5 b (1 NEX)	16 b (1 NEX) vs. 5 b (3 NEX)	5 b (1 NEX) vs. 5 b (3 NEX)
**ADC**_**0**_	0.80 (0.37–0.94)	0.70 (0.02–0.91)	0.84 (0.48–0.95)
**K**	0.91 (0.71–0.97)	0.87 (0.59–0.96)	0.91 (0.72–0.97)
**sADC**	0.80 (0.32–0.94)	0.84 (0.45–0.95)	0.85 (0.52–0.96)

Data are mean values with 95% confidence intervals in parentheses.

ICCs of perfusion parameters between different number of b-values or excitations were not estimated, due to large coefficients of variations.

**Table 6:** Intraclass correlation coefficient of diffusion parameters in malignant and benign breast tumor datasets using one and three number of excitations (NEX) of five b-values.

Parameters	malignant	benign
**ADC**_**0**_	0.99 (0.95–1.00)	0.91 (0.60–0.98)
**K**	0.94 (0.70–0.99)	1.00 (1.00–1.00)
**sADC (10**^**−3**^ **mm**^**2**^/s)*	0.99 (0.97–1.00)	0.97 (0.85–0.99)

Data are mean values with 95% confidence intervals in parentheses.

ICCs of perfusion parameters between different number of b-values or excitations were not estimated, due to large coefficients of variations.

Almost perfect agreement was observed in diffusion and perfusion parameters between one and three NEX of 5 b values in malignant and benign breast lesions, (Table 6).

**Fig 3:**
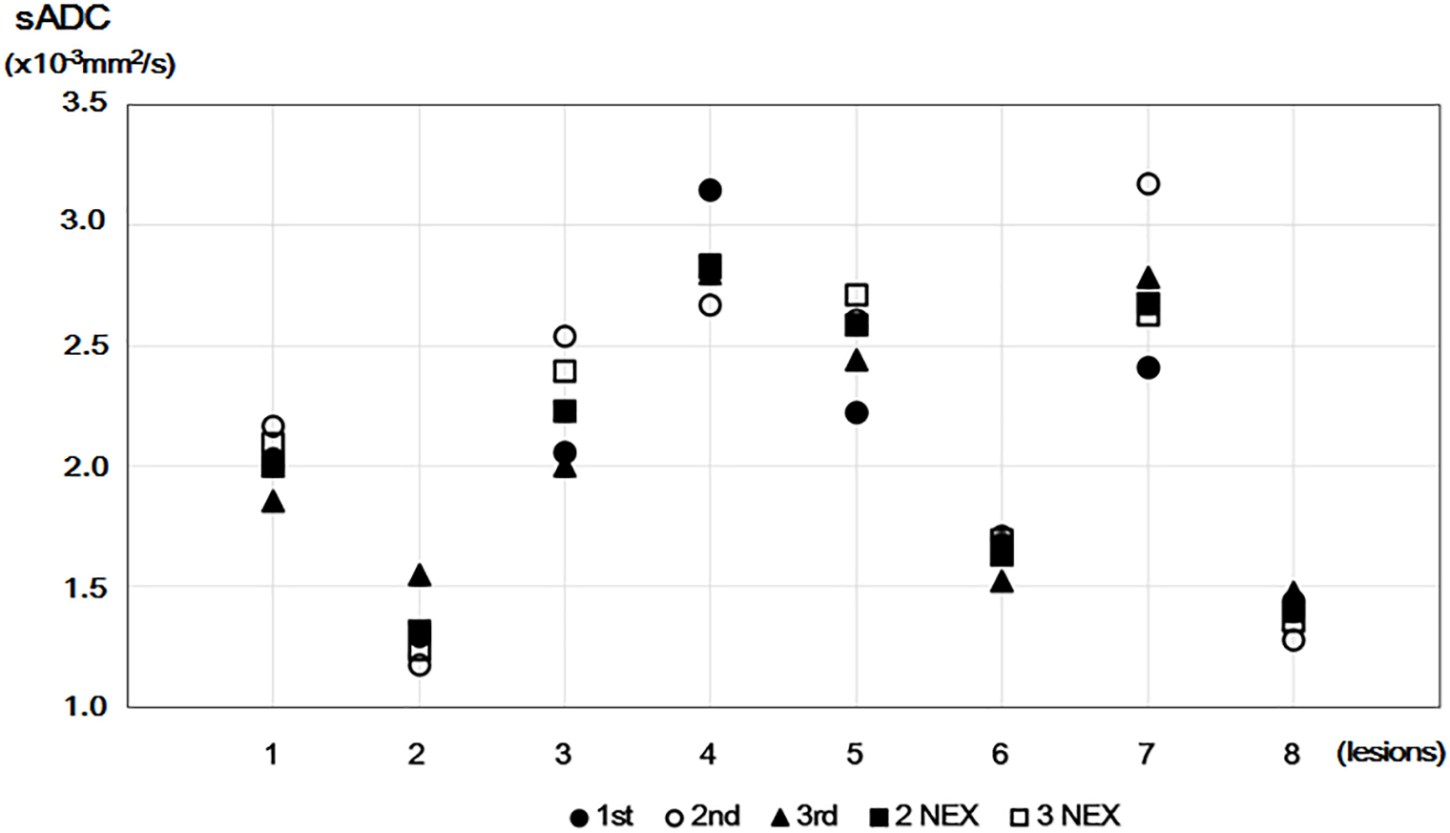
Plots of sADC values in benign lesions using five b-values. Plots of sADC values obtained using one NEX (1st, 2nd, and 3rd), two NEX, and three NEX from five b-value DWI datasets for eight benign breast tumors.

Plots of sADC values in benign lesions using five b-value DWI datasets are shown in Fig 3. Several plots (even with averaging) deviated from the other plots. For example, sADC values in the plots with two and three NEX were different because of deviation in the plot of the second acquisition in case 3.

## Discussion

In the present study, we examined the changes in behavior of diffusion and perfusion parameters from different diffusion MR acquisition schemes in normal breast tissue, and in malignant and benign breast tumors. Improvements in the gradient hardware of recent MR systems has allowed data to be obtained at higher b-values, allowing new estimates of IVIM and non-Gaussian diffusion MR parameters beyond ADC [9]. Several studies have examined the diagnostic performance of IVIM [8, 21, 22], diffusion kurtosis imaging [12, 13], and hybrid diffusion kurtosis imaging/IVIM [4] in the breast, However, their reliability is unknown, and is important to investigate prior to clinical application. An advantage of diffusion MRI is that contrast agents are not required, which is important given the discovery that gadolinium can accumulate in tissues, particularly in the brain [23, 24]. There is also growing interest in the use of DWI as an alternative to contrast-enhanced MRI for breast cancer screening, although validation of the parameters obtained from DWI with multiple b-values is required [25]. In the present study, we provide new data on the variability of IVIM and non-Gaussian diffusion parameters with different NEX in normal breast tissue.

There were no statistical differences in diffusion and perfusion parameters in normal breast or breast lesions tissue, regardless of the number of b-values or excitations used. The wCVs for diffusion coefficients between the different diffusion acquisition methods were 1.4%–2.7% in normal breast tissues or breast lesions, smaller than the reported wCV for ADC (11%) [20] or scanner stability (6.6%) and scan-scan reproducibility (8%) for ADC [26]. This small wCV may be related to our use of consecutive scans, while the above studies examined variability or reproducibility of ADC values by scanning volunteers twice on different days. These data also indicate that different combinations of b-values or NEX did not significantly influence the variability or reproducibility of the diffusion parameters.

The ICCs of the diffusion parameters comparing different b-values or NEX were generally substantial and almost perfect, suggesting that there was no significant effect of different combinations of b-values or NEX on the reliability of the diffusion parameters. By contrast, the ICCs of the perfusion parameters showed poor agreement among different b-values or NEX. Thus, precise estimation of IVIM parameters is challenging in low-perfused tissue such as normal breast tissue, and further improvements are required (e.g., improving the SNR, averaging, or new models) for precise IVIM estimation in low-perfused tissue [27].

A limited protocol using only five b-values may be useful in the clinical setting, resulting in a significant reduction in acquisition time. Alternatively, an sADC calculated from only two values may provide data encompassing both Gaussian and non-Gaussian diffusion effects [9]. However, protocols with more b-values may be required in cases of noisy datasets, or for improving the accuracy of estimated non-Gaussian and IVIM MRI parameters. In addition, improvement of noise handling or better SNR at high b-values using a non-Gaussian DWI model will improve the estimation of fIVIM and D*.

Signal averaging is expected to increase SNR. Indeed, we observed a decrease in the CVs of ADC_0_ values with the increase in the number of NEX in a nonane phantom, while the mean value remained stable. However, this trend was not found in normal breast tissue or breast lesions, which is counterintuitive. The possible explanation can be found in Fig 3, which indicates the presence of outliers in some subjects. The outliers likely result from motion, which often occurs in a clinical setting, particularly with poorly cooperative patients. As the presence of outliers may increase the standard-deviation and cause erroneous mean values, it is important to check for outliers before signal averaging. By contrast, there were no statistical differences in IVIM or non-Gaussian diffusion parameter estimates when using 16 b-values (with one NEX) or five b-values (with three NEX) for fitting. Thus, a five b-value protocol may be suitable for evaluation of breast lesions, providing the SNR is sufficiently high after averaging. Note that the total acquisition times for 16 b-values (with one NEX) or five b-values (with three NEX) were similar.

The perfusion parameters including fIVIM exhibited large standard deviations, and sufficient agreement was not expected. The poor perfusion in normal breast tissue compared with malignant or benign lesions [4] make the fitting process challenging, which is a limitation of IVIM.

Other limitations of our study include the small number of volunteers and patients. The reproducibility, repeatability, and diagnostic accuracy of ADC values in breast lesions were previously reported [28], indicating almost perfect agreement, and which were higher than those in normal breast tissue [26]. The higher SNR in breast lesions compared with normal tissue may result in reduced variation in ADC values.

In conclusion, we investigated the variability of non-Gaussian diffusion MRI measurements on different numbers of b-values and excitations in normal breast tissue and breast lesions. We found no statistical differences between non-Gaussian MRI parameters in normal breast tissue and breast lesions regardless of the number of b-values or excitations used.

## Supporting information

S1 Fig Comparison of ADC_0_ values in a nonane phantom. Four different diffusion weighted imaging (DWI) datasets were analyzed using five b-values each for one number of excitations (NEX), two NEX, and three NEX. Mean and standard deviations are shown as lines.
(TIF)

S1 Table Diffusion and perfusion parameters in normal breast tissues. (XLSX)
