# Supplementary figures and images for: Variability of non-Gaussian diffusion MRI and intravoxel incoherent motion (IVIM) measurements in the breast
Source: PLoS One. 2018 Mar 1;13(3):e0193444. doi: 10.1371/journal.pone.0193444 (PMC5832256; doi:10.1371/journal.pone.0193444)

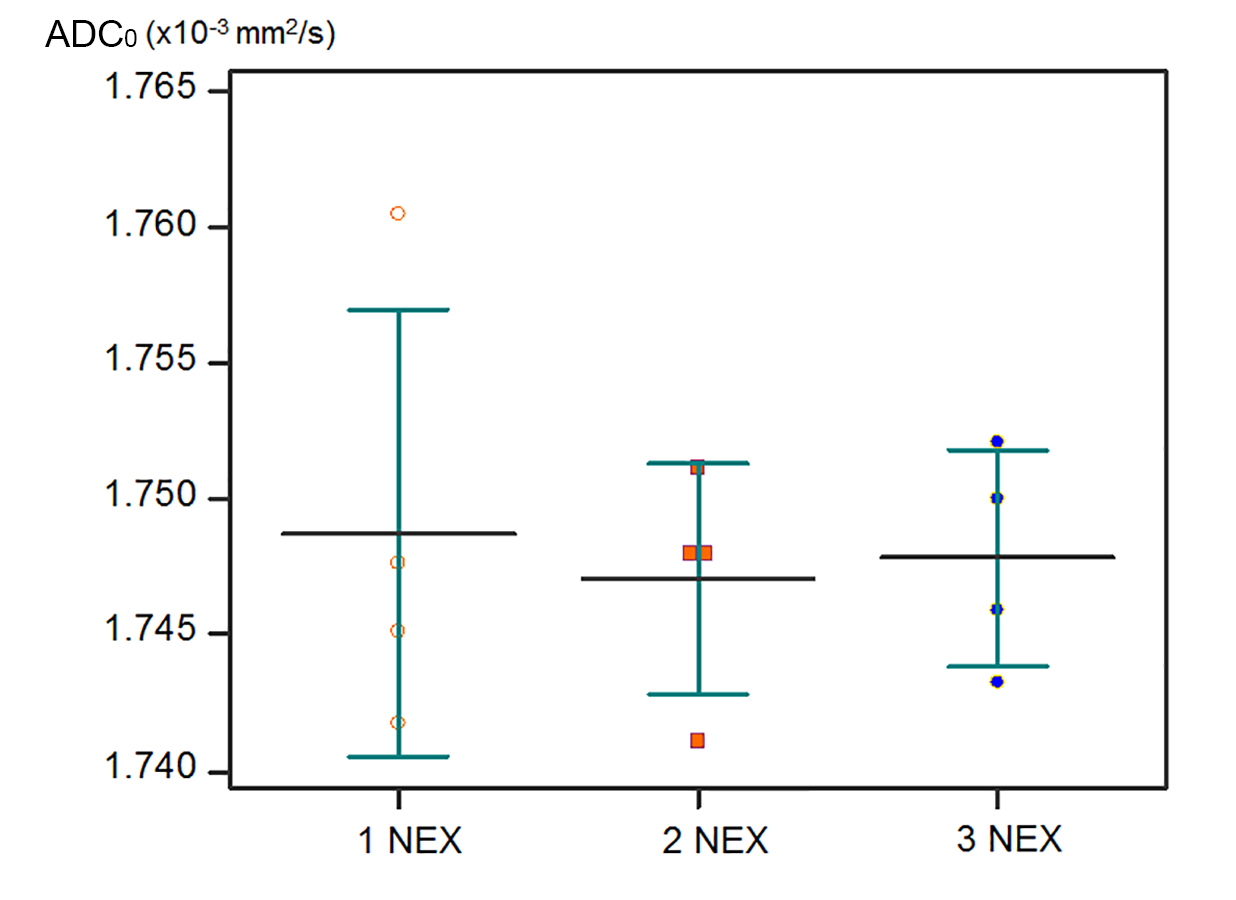

Supplement: S1 Fig — Four different diffusion weighted imaging (DWI) datasets were analyzed using five b-values each for one number of excitations (NEX), two NEX, and three NEX. Mean and standard deviations are shown as lines. (TIF) [file pone.0193444.s001.tif]
